# Gaming behavior disorder and its association with social phobia during COVID-19 pandemic: A cross-sectional study among the young Arabs

**DOI:** 10.3389/fpsyt.2023.1071764

**Published:** 2023-04-11

**Authors:** Nour Shaheen, Ahmed Shaheen, Mohamed Elmasry, Omar Ahmed Abdelwahab, Abdelrahman Mohamed, Sarya Swed, Ala’ Abdala Rababah, Mostafa Meshref, Ahmed Zaki, Sheikh Shoib

**Affiliations:** ^1^Alexandria Faculty of Medicine, Alexandria University, Alexandria, Egypt; ^2^Faculty of Medicine, Al-Azhar University, Cairo, Egypt; ^3^Medical Research Group of Egypt, Cairo, Egypt; ^4^Faculty of Medicine, Aleppo University, Aleppo, Syria; ^5^King Hussein Medical Center, Internal Medicine, Amman, Jordan; ^6^Neurology Department, Faculty of Medicine, Al-Azhar University, Cairo, Egypt; ^7^Faculty of Medicine, Cairo University, Cairo, Egypt; ^8^Department of Health Services, Kashmir, India; ^9^Sharda University, Greater Noida, Uttar Pradesh, India; ^10^Mind Wellness Center, Nawab Bazar Srinagar, India

**Keywords:** Online Gaming Behavior Disorder, social phobia disorder, gaming behavior, COVID-19 pandemic, DSM-5, Internet Gaming Disorder (IGD)

## Abstract

**Background:**

Gaming addiction is a compulsive mental health condition that can have severe negative consequences on a person’s life. As online gaming has increased during the COVID-19 pandemic, studies have shown a heightened risk of mental health issues. This study aims to assess the prevalence of severe phobia and addiction to online gaming among Arab adolescents and identify risk factors associated with these disorders.

**Methods:**

This cross-sectional study was conducted across 11 Arab nations. Participants were recruited using convenience sampling through an online survey distributed on social media platforms in 11 Arab countries. The survey included demographic questions, the Nine-item Internet Gaming Disorder Scale-Short Form (IGDS-SF9) to measure participants’ online gaming addiction, the Social Phobia Scale (SPS), and questions assessing the impact of the COVID-19 pandemic on the prevalence of internet gaming addiction. The data were analyzed using SPSS win statistical package version 26.

**Results:**

Out of 2,458 participants, 2,237 were included in the sample due to non-response and missing data. The average age of the participants was 19.9 ± 4.8 years, and the majority were Egyptian and unmarried. 69% of the participants reported playing more than usual since the COVID-19 pandemic, as they were confined to their homes. Higher social phobia scores were associated with being single, male, and Egyptian. Participants from Egypt and those who felt that the pandemic significantly increased their gaming time had higher scores for online gaming addiction. Several major criteria, such as playing hours per day and beginning gaming at an early age, were associated with a higher level of online gaming addiction with social phobia.

**Conclusion:**

The study’s findings suggest that there is a high prevalence of internet gaming addiction among Arab adolescents and young adults who play online games. The results also indicate a significant association between social phobia and several sociodemographic factors, which may inform future interventions and treatments for individuals with gaming addiction and social phobia.

## 1. Introduction

The COVID-19 pandemic has a significant impact on various aspects of human life worldwide, including psychological health, as reported in almost all countries (1, 2). The Arab nations are among the populations affected by poor mental health (3, 4). As of June 2020, the Centers for Disease Control and Prevention (CDC) reported that approximately one-third of individuals in the US suffer from depression or anxiety (5). Among young people, the incidence of mental health issues has increased dramatically over the last decade, with nearly 60% of those between the ages of 18 and 24 considered at risk for anxiety or depression, and 25% expressing suicidal ideation in the past month, compared to 11% of all individuals in 2019 (6, 7). These rates represent a substantial increase in depression, coinciding with the implementation of stay-at-home orders, campus closures, and social distancing measures, which have significantly disrupted daily life and interactions (8, 9).

To mitigate the impact of the pandemic on mental health, the World Health Organization (WHO) has recommended video games as part of its “Healthy at Home” program (10). However, excessive gaming may pose a risk to vulnerable individuals, including minors and those with gaming disorders (11). The pandemic has made it difficult to detect harmful effects such as harm to mental health or sleep patterns, as work and school have shifted to home-based practices (12).

Moreover, prolonged stress during the pandemic may lead to significant emotional distress, including anxiety and depression (13, 14). Although online games can help individuals deal with unpleasant emotions, greater involvement may make them more susceptible to Internet Gaming Disorder (IGD) (12, 15, 16). According to the Diagnostic and Statistical Manual of Mental Disorders, 5th edition (DSM-5) criteria, IGD is characterized by excessive and prolonged internet gaming, which leads to behavioral and cognitive symptoms such as progressive loss of control, tolerance, and withdrawal symptoms, similar to substance use disorders (17). IGD is associated with poorer mental health, sleep quality, interpersonal problems, and academic achievement, and is linked to lower quality of life (18).

This aim of this study is to assess the impact of the COVID-19 pandemic on the prevalence of IGD and SPS among online gamers in the Arab world as well as dentifying risk factors for IGD negative consequences associated with these disorders.

## 2. Methods

### 2.1. Study design, setting

This study aimed to assess the prevalence of internet gaming addiction (IGA) and its association with social phobia among Arab adolescents and young adults aged 15 to 30 who played online games. The cross-sectional study was conducted between September 2021 and March 2022 in 11 Arab countries, namely Egypt, Syria, Palestine, Jordan, Tunisia, Iraq, Morocco, Algeria, Sudan, Libya, and Lebanon.

### 2.2. Participants

Convenience sampling, a non-probability sampling method, was used to collect data from responders. Participants were recruited through an online survey using Google Forms, which was placed on Arab online gaming and social forums. The survey was designed to be completed in approximately 10 min and distributed on social media platforms such as Facebook, WhatsApp, Twitter, and Telegram to reach a substantial sample size.

The questionnaire included three sections: the first section gathered information on the participants’ demographics, the second section used the Nine-item Internet Gaming Disorder Scale-Short Form (IGDS-SF9) to measure participants’ online gaming addiction, and the third section included the Social Phobia Scale (SPS) and three questions assessing the impact of the COVID-19 pandemic on the prevalence of internet gaming addiction.

### 2.3. Measurements

The first section of the questionnaire gathered information on the participants’ demographics, including age, sex, marital status, level of education, drug use history, level of smoking, country of residence, and gaming status.

The Nine-item Internet Gaming Disorder Scale-Short Form (IGDS-SF9) was used to measure participants’ online gaming addiction. The scale consists of nine items and assesses the symptoms of internet gaming addiction, including salience, mood modification, tolerance, withdrawal, conflict, relapse, problems, deception, and loss of control. Participants rated each item on a five-point Likert scale ranging from 1 (never) to 5 (very often). The scores ranged from 9 to 45, with higher scores indicating a higher degree of addiction (19).

The Social Phobia Scale (SPS) was used to measure social phobia. The SPS is a 20-item self-report measure that assesses the fear and avoidance of social situations. Participants rated each item on a five-point Likert scale ranging from 0 (not at all) to 4 (extremely). The scores ranged from 0 to 80, with higher scores indicating a higher degree of social phobia (20).

### 2.4. Sample size

The sample size for this study was determined using the formula described by Swinscow and Cohen^1^ [*n* = [(Za/2)2. P (1-P)]/d2]. Assuming a prevalence of 50% in the population, a confidence level of 95%, a margin of error of 5%, and a 5% addition for the non-response rate, the recommended sample size was 384 individuals. A total of 2,458 people participated in the survey. After excluding 76 participants who declined to complete the survey and 145 instances of missing data, the final sample size was 2,237.

### 2.5. Pilot study

To ensure the survey’s comprehensibility and to avoid errors, a pilot test was conducted with 50 participants. Cronbach’s alpha values indicated strong internal consistency for the IGA and SPS scales (0.85 and 0.84, respectively).

### 2.6. Statistical analysis

The data were stored in an Excel sheet and analyzed using SPSS win statistical package version 26. Numerical data were summarized as means and standard deviations or medians and ranges as appropriate. Qualitative data were described as frequencies and percentages. Comparison between two groups for numerical variables was done using either the student t-test or the Mann–Whitney *U* test (non-parametric *t*-test) as appropriate. Comparison between more than two groups for numerical variables was done using ANOVA or non-parametric Kruskal-Wallis as appropriate. The relationship between qualitative data was analyzed using the Chi-square test or Fisher’s exact test as appropriate. Binary logistic regression was used to determine the potential association between the risk of developing a high degree of internet gaming addiction (IGA score >) and social phobia (SPS score >), based on the participants’ baseline characteristics.

## 3. Results

### 3.1. Descriptive characteristics of sample by sociodemographic factors

In this study, 2,237 individuals were included in the analysis, with a majority being male (2,133 individuals), and a mean age of 19.9 years. Most of the participants were from Egypt (76.7%), single (87.5%), and 2,102 of them were gamers. The study of gamers revealed that 73.8% played for less than 5 hours per day, and the majority (84%) started gaming before the age of 12 years. Approximately 75% of gamers reported watching streamers, with 35% indicating they watched “a lot.” Since the COVID-19 pandemic, 69% of participants reported playing more than usual since they were unable to leave their homes (Table 1).

**Table 1:** Baseline characteristics of the study sample.

Variable		Frequency (Percentage)
Age (Mean ± SD)		19.9 ± 4.8
Gender	Male	2,133 (95.4)
	Female	104 (4.6)
Marital status	Single	1958 (87.5)
	Engaged	183 (8.2)
	Married	87 (3.9)
	Divorced	9 (0.4)
Education Status	University education or after university	1,281 (57.3)
	Secondary certificate or technical education	606 (27.1)
	Middle school	330 (14.8)
	Other	16 (0.7)
	Uneducated	2 (0.1)
	Secondary certificate and technical education	1 (0.0)
	Elementary school	1 (0.0)
Drug History	Not at all	2010 (89.9)
	I was taking in the past and I no longer use it now	88 (3.9)
	other	86 (3.8)
	I am taking now, but not abundant	39 (1.7)
	I am currently using drugs	12 (0.5)
	I am trying to take off now or under a rehabilitation program	2 (0.1)
Smoking Status	No	1834 (82.0)
	Yes	264 (11.8)
	Past smoker	139 (6.2)
Country	Egypt	1716 (76.7)
	Syria	150 (6.7)
	Palestine	71 (3.2)
	Jordan	60 (2.7)
	Tunisia	43 (1.9)
	Iraq	43 (1.9)
	Morocco	33 (1.5)
	Algeria	27 (1.2)
	Sudan	26 (1.2)
	Libya	21 (0.9)
	Lebanon	21 (0.9)
	Other	21 (0.9)
Gamer	yes	2,102 (94.0)
	no	135 (6.0)

### 3.2. Relationships between the social phobia scale and different sociodemographic factors

The study found that social phobia scores were significantly associated with several sociodemographic factors, including national origin, gender, marital status, education status, age of first gameplay, gaming hours per week, COVID-19 impact on gaming attitudes, and increased dependence on gaming due to isolation (*p* < 0.001). Being single, Egyptian, or male was associated with higher social phobia scores. Education level, age of first gameplay, and gaming hours also demonstrated significant differences (p < 0.001). Additionally, the study found a significant difference in self-reported changes in gaming behavior during the pandemic (*p* < 0.004), with those who reported “significantly increased” playing having a slightly higher SPS score than those whose behavior did not change (Table 2).

**Table 2:** SPS score and sociodemographic characteristics.

Variable	Category	*N* %	Mean + SD	*p* value
Gender	Male	2043 (97.2)	18.00 ± 4.86	<0.001
	Female	59 (2.8)	17.49 ± 5.43	
Marital status	Single	1851 (88.1)	18.03 ± 4.83	<0.001
	Engaged	169 (8.0)	17.58 ± 4.79	
	Married	74 (3.5)	17.61 ± 5.37	
	Divorced	8 (0.4)	17.88 ± 8.72	
Education Status	University education or after university	1,183 (56.3)	17.98 ± 4.93	0.014
	Secondary certificate or technical education	579 (27.5)	17.99 ± 4.83	
	Middle school	321 (15.3)	17.98 ± 4.66	
	Other	15 (0.7)	19.27 ± 4.85	
	Uneducated	2 (0.1)	18.00 ± 9.00	
	Elementary school	1 (0.0)	11.00 ± 0.00	
Drug History	Not at all	1889 (89.9)	17.97 ± 4.87	0.827
	I was taking in the past and I no longer use it now	84 (4.0)	17.89 ± 4.75	
	other	80 (3.8)	18.21 ± 4.82	
	I am taking now, but not abundant	38 (1.8)	18.76 ± 4.69	
	I am currently using drugs	9 (0.4)	15.00 ± 6.13	
	I am trying to take off now or under a rehabilitation program	2 (0.1)	19.50 ± 1.50	
Smoking Status	No	1723 (82.0)	18.00 ± 4.87	0.165
	Yes	245 (11.7)	17.49 ± 5.03	
	Past smoker	134 (6.4)	18.59 ± 4.59	
Starting Gaming Age	Between 7 and 12 years	984 (46.8)	18.06 ± 4.88	0.014
	Before the age of six	721 (34.3)	17.76 ± 4.74	
	Between 13 and 17 years	276 (13.1)	18.41 ± 4.76	
	I do not remember well	62 (2.9)	18.19 ± 5.19	
	Between 18 years 30 years	59 (2.8)	17.20 ± 6.11	
Playing Hours per hour	From three to five hours	950 (45.2)	18.02 ± 4.60	0.001
	Two hours	449 (21.4)	17.15 ± 4.58	
	From five to ten hours	445 (21.2)	19.59 ± 5.15	
	hour	111 (5.3)	16.07 ± 4.42	
	More than ten hours	106 (5.0)	17.64 ± 5.52	
	half an hour	41 (2.0)	14.71 ± 4.57	
Covid-19 impact on gaming attitude	Significantly increased	740 (35.2)	18.56 ± 5.01	0.004
	Slightly increased	710 (33.8)	18.00 ± 4.70	
	It remained as it was before the epidemic	563 (26.8)	17.23 ± 4.87	
	Slightly decreased	89 (4.2)	17.74 ± 4.38	
Isolation increased dependence on gaming	Entirely Agree	869 (41.3)	18.92 ± 4.88	<0.001
	Rather Agree	678 (32.3)	17.74 ± 4.60	
	Rather Disagree	236 (11.2)	16.31 ± 4.61	
	I do not know	222 (10.6)	18.03 ± 4.80	
	Entirely Disagree	97 (4.6)	15.15 ± 5.07	
Gaming as a source of entertainment during pandemic	Entirely Agree	1,564 (74.4)	18.02 ± 4.75	0.527
	Rather Agree	428 (20.4)	17.73 ± 5.13	
	I do not know	64 (3.0)	19.17 ± 5.36	
	Rather Disagree	33 (1.6)	17.94 ± 5.33	
	Entirely Disagree	13 (0.6)	16.23 ± 5.65	
Country	Egypt	1,609 (76.5)	18.25 ± 4.88	0.004
	Syria	145 (6.9)	17.77 ± 4.54	
	Palestine	64 (3.0)	17.59 ± 5.05	
	Jordan	57 (2.7)	16.40 ± 4.10	
	Tunisia	42 (2.0)	17.00 ± 4.86	
	Iraq	41 (2.0)	17.80 ± 4.10	
	Morocco	31 (1.5)	16.94 ± 4.85	
	Algeria	26 (1.2)	16.00 ± 4.51	
	Sudan	25 (1.2)	16.08 ± 6.46	
	Libya	21 (1.0)	17.10 ± 4.93	
	Lebanon	20 (1.0)	16.25 ± 4.87	
	Other	21 (0.9)	15.2 ± 3.77	
Following Streamers	Missing	806 (38.3)	17.84 ± 4.82	0.653
	Yes, a little	737 (35.1)	18.17 ± 4.84	
	Yes, a lot	346 (16.5)	18.61 ± 4.88	
	not at all	213 (10.1)	16.86 ± 4.93	

### 3.3. Relationships between internet gaming disorder scores and different sociodemographic factors

The study found that gender, marital status, education level, and age at which the first game was played had no effect on scores for Internet Gaming Disorder (IGD). However, the study did observe significant differences in IGD scores based on nationality (*p* < 0.003), changes in play behavior during the pandemic (*p* < 0.001), duration of gaming per day (*p* < 0.001), and following streamers (*p* < 0.001). Specifically, participants who played games for five to 10 hours per day had scores almost 5 points higher on the IGD scale compared to those who played for half an hour. Moreover, gamers who watched “a lot” of streamers had scores two points higher on the IGD scale than those who did not watch. Additionally, Egyptian participants and those who reported a significant increase in gaming time due to the COVID-19 epidemic had the highest IGD scores (Table 3).

**Table 3:** IGD score and sociodemographic characteristics.

Variable	Category	*N* %	Mean + SD	*p*-value
Gender	Male	2043 (97.2)	18.00 ± 4.86	0.434
	Female	59 (2.8)	17.49 ± 5.43	
Marital status	Single	1851 (88.1)	18.03 ± 4.83	0.616
	Engaged	169 (8.0)	17.58 ± 4.79	
	Married	74 (3.5)	17.61 ± 5.37	
	Divorced	8 (0.4)	17.88 ± 8.72	
Education status	University education or after university	1,183 (56.3)	17.98 ± 4.93	0.371
	Secondary certificate or technical education	579 (27.5)	17.99 ± 4.83	
	Middle school	321 (15.3)	17.98 ± 4.66	
	Other	15 (0.7)	19.27 ± 4.85	
	Uneducated	2 (0.1)	18.00 ± 9.00	
	Elementary school	1 (0.0)	11.00 ± 0.00	
Drug history	Not at all	1889 (89.9)	17.97 ± 4.87	0.447
	I was taking in the past and I no longer use it now	84 (4.0)	17.89 ± 4.75	
	other	80 (3.8)	18.21 ± 4.82	
	I am taking now, but not abundant	38 (1.8)	18.76 ± 4.69	
	I am currently using drugs	9 (0.4)	15.00 ± 6.13	
	I am trying to take off now or under a rehabilitation program	2 (0.1)	19.50 ± 1.50	
Smoking status	No	1723 (82.0)	18.00 ± 4.87	0.099
	Yes	245 (11.7)	17.49 ± 5.03	
	Past smoker	134 (6.4)	18.59 ± 4.59	
Starting gaming age	Between 7 and 12 years	984 (46.8)	18.06 ± 4.88	0.238
	Before the age of six	721 (34.3)	17.76 ± 4.74	
	Between 13 and 17 years	276 (13.1)	18.41 ± 4.76	
	I do not remember well	62 (2.9)	18.19 ± 5.19	
	Between 18 years 30 years	59 (2.8)	17.20 ± 6.11	
Playing hours per day	From three to five hours	950 (45.2)	18.02 ± 4.60	<0.001
	Two hours	449 (21.4)	17.15 ± 4.58	
	From five to ten hours	445 (21.2)	19.59 ± 5.15	
	hour	111 (5.3)	16.07 ± 4.42	
	More than ten hours	106 (5.0)	17.64 ± 5.52	
	half an hour	41 (2.0)	14.71 ± 4.57	
Covid-19 impact on gaming attitude	Significantly increased	740 (35.2)	18.56 ± 5.01	<0.001
	Slightly increased	710 (33.8)	18.00 ± 4.70	
	It remained as it was before the epidemic	563 (26.8)	17.23 ± 4.87	
	Slightly decreased	89 (4.2)	17.74 ± 4.38	
Isolation increased dependence on gaming	Entirely Agree	869 (41.3)	18.92 ± 4.88	<0.001
	Rather Agree	678 (32.3)	17.74 ± 4.60	
	Rather Disagree	236 (11.2)	16.31 ± 4.61	
	I do not know	222 (10.6)	18.03 ± 4.80	
	Entirely Disagree	97 (4.6)	15.15 ± 5.07	
Gaming as a source of entertainment during pandemic	Entirely Agree	1,564 (74.4)	18.02 ± 4.75	0.152
	Rather Agree	428 (20.4)	17.73 ± 5.13	
	I do not know	64 (3.0)	19.17 ± 5.36	
	Rather Disagree	33 (1.6)	17.94 ± 5.33	
	Entirely Disagree	13 (0.6)	16.23 ± 5.65	
Country	Egypt	1,609 (76.5)	18.25 ± 4.88	0.003
	Syria	145 (6.9)	17.77 ± 4.54	
	Palestine	64 (3.0)	17.59 ± 5.05	
	Jordan	57 (2.7)	16.40 ± 4.10	
	Tunisia	42 (2.0)	17.00 ± 4.86	
	Iraq	41 (2.0)	17.80 ± 4.10	
	Morocco	31 (1.5)	16.94 ± 4.85	
	Algeria	26 (1.2)	16.00 ± 4.51	
	Sudan	25 (1.2)	16.08 ± 6.46	
	Libya	21 (1.0)	17.10 ± 4.93	
	Lebanon	20 (1.0)	16.25 ± 4.87	
	Other	21 (0.9)	15.2 ± 3.77	
Following streamers	Missing	806 (38.3)	17.84 ± 4.82	<0.001
	Yes, a little	737 (35.1)	18.17 ± 4.84	
	Yes, a lot	346 (16.5)	18.61 ± 4.88	
	not at all	213 (10.1)	16.86 ± 4.93	

### 3.4. Potential predictors of a high level of IGD & SPS scores

Of the nine variables examined in this study, only two were found to be statistically significant predictors of high levels of internet addiction: daily playing hours and starting age of gaming. Specifically, individuals who spent half to 2 hours per day on the internet were less likely to have a high level of internet gaming addiction compared to those who spent 5 to 10 h per day. Conversely, those who started gaming between the ages of seven and seventeen were more likely to have a high level of internet gaming addiction than those who began gaming before the age of six.

Six factors were found to be statistically significant predictors of high levels of social phobia: gender, nationality, the impact of Covid-19 on gaming attitudes, social status, starting age of gaming, and daily playing hours. Males were less likely to have a high level of social phobia than females. Divorced individuals were 3.527 times more likely to experience severe social phobia than single individuals. Furthermore, those who spent 2 hours on internet gaming were less likely to experience (Table 4).

**Table 4:** Binary logistic regression for prediction of the high level of social phobia, and internet gaming disorder.

Variable	Category	*N* (%)	High Level of Social Phobia				High Risk of Internet Gaming Disorder			
			OR	95% CI		*p* value	OR	96% CI		*p* value
				Lower	Upper			Lower	Upper	
Gender	Female	59 (0.03%)	REF							
	Male	2043 (0.97%)	0.314	0.186	0.532	<0.001	1.090	0.584	2.034	0.787
Social Status	Single	1851 (0.88%)	REF							
	Engaged	169 (0.08%)	0.883	0.454	0.948	0.025	−1.307	0.602	1.297	0.528
	Married	74 (0.04%)	1.209	0.101	0.485	<0.001	−0.993	0.716	2.043	0.477
	Divorced	8 (0.00%)	3.527	0.840	14.807	0.085	0	0.813	13.109	0.095
Drug History	Other	80 (0.04%)	REF							
	I am currently using drugs	9 (0.00%)	1.662	0.412	6.708	0.476	0.310	0.037	2.619	0.282
	I am taking now, but not abundant	38 (0.02%)	0.846	0.364	1.966	0.698	1.144	0.495	2.644	0.753
	I am trying to take off now or under a rehabilitation program	2 (0.00%)	0.000	0.000	inf	1.000	0.000	0.000	inf	1.000
	I was taking in the past and I no longer use it now	84 (0.04%)	0.880	0.454	1.705	0.705	0.583	0.281	1.208	0.147
	Not at all	1889 (0.90%)	0.920	0.571	1.484	0.733	0.757	0.461	1.243	0.271
Smoking Status	No	1723 (0.82%)	REF							
	Past Smoker	134 (0.06%)	0.762	0.512	1.135	0.182	1.106	0.738	1.658	0.624
	Yes	245 (0.12%)	0.778	0.576	1.052	0.103	0.942	0.684	1.297	0.715
Starting Gaming Age	Before the age of six	721 (0.34%)	REF							
	Between 13 and 17 years	276 (0.13%)	0.814	0.596	1.110	0.193	1.443	1.045	1.992	0.026
	Between 18 years 30 years	59 (0.03%)	0.842	0.464	1.527	0.571	1.225	0.655	2.293	0.525
	Between 7 and 12 years	984 (0.47%)	1.031	0.837	1.269	0.775	1.270	1.007	1.603	0.044
	I do not remember well	62 (0.03%)	1.863	1.103	3.149	0.020	1.875	1.068	3.292	0.028
Playing Hours per Day	From five to ten hours	445 (0.21%)	REF							
	From three to five hours	950 (0.45%)	0.899	0.707	1.143	0.385	0.549	0.429	0.704	<0.001
	More than ten hours	106 (0.05%)	1.304	0.843	2.017	0.233	0.678	0.422	1.090	0.108
	Two hours	449 (0.21%)	0.676	0.506	0.903	0.008	0.429	0.315	0.583	<0.001
	half an hour	41 (0.02%)	0.922	0.464	1.833	0.817	0.204	0.071	0.584	0.003
	hour	111 (0.05%)	0.805	0.510	1.269	0.350	0.208	0.108	0.399	<0.001
Covid-19 impact on gaming attitude	It remained as it was before the epidemic	563 (0.27%)	REF							
	Significantly increased	740 (0.35%)	1.451	1.146	1.838	0.002	1.650	1.269	2.147	<0.001
	Slightly increased	710 (0.34%)	0.925	0.723	1.184	0.538	1.192	0.907	1.568	0.208
	Slightly decreased	89 (0.04%)	0.771	0.457	1.300	0.329	0.983	0.557	1.736	0.954
Country	Jordan	57 (0.03%)	5.333	0.649	43.854	0.119	4.255	0.510	35.494	0.181
	Algeria	26 (0.01%)	10.588	1.215	92.253	0.033	2.609	0.251	27.113	0.422
	Sudan	25 (0.01%)	7.778	0.871	69.490	0.066	7.778	0.871	69.490	0.066
	Iraq	41 (0.02%)	9.286	1.122	76.847	0.039	5.625	0.662	47.818	0.114
	Morocco	31 (0.01%)	12.632	1.494	106.766	0.020	3.846	0.416	35.582	0.235
	Tunisia	42 (0.02%)	5.455	0.642	46.330	0.120	3.333	0.374	29.678	0.280
	Syria	145 (0.07%)	6.126	0.793	47.339	0.082	5.217	0.673	40.454	0.114
	Palestine	64 (0.03%)	7.826	0.977	62.705	0.053	6.122	0.757	49.499	0.089
	Lebanon	20 (0.01%)	5.000	0.507	49.266	0.168	2.222	0.185	26.629	0.529
	Libya	21 (0.01%)	6.250	0.662	59.027	0.110	6.250	0.662	59.027	0.110
	Egypt	1,609 (0.77%)	9.604	1.285	71.758	0.027	6.639	0.888	49.626	0.065
	Other	21 (0.01%)	REF							
Following Streamers	Yes, a little	737 (0.35%)	REF							
	Yes, a lot	346 (0.16%)	1.155	0.878	1.519	0.304	1.129	0.842	1.513	0.417
	not at all	213 (0.10%)	1.078	0.776	1.499	0.653	0.800	0.550	1.164	0.244

## 4. Discussion

The present study investigated the relationship between sociodemographic factors and both Internet Gaming Disorder (IGD) and Social Phobia Scale (SPS) scores among gamers. The findings suggest that sociodemographic factors play an important role in predicting levels of IGD and SPS, indicating the complex and multifaceted nature of these conditions.

In terms of IGD, the study found that daily playing hours and following streamers were significant predictors of high levels of internet addiction. These findings suggest that certain behaviors, such as excessive gaming or following online influencers, may lead to increased risk of developing IGD. It is important to note that excessive gaming has been associated with a range of negative outcomes, including poor academic performance, social isolation, and decreased physical activity. As such, clinicians should be aware of the potential risks associated with excessive gaming and work with patients to establish healthy gaming habits.

The study also found that sociodemographic factors, such as nationality and the impact of COVID-19 on gaming attitudes, were significant predictors of IGD. Specifically, individuals from Egypt and those who reported a significant increase in gaming time due to the pandemic had the highest IGD scores. The COVID-19 pandemic has led to significant changes in daily routines and social interactions, and as such, it is not surprising that there has been an increase in gaming activity. However, clinicians should be aware of the potential negative impact of increased gaming on mental health and wellbeing.

In terms of SPS, the study found that gender, nationality, and daily playing hours were significant predictors of high levels of social phobia. These findings suggest that there may be a complex interplay between gaming behavior, social anxiety, and sociodemographic factors. Social phobia can be a debilitating condition that can significantly impact an individual’s quality of life. As such, clinicians should be aware of the potential risk factors associated with social phobia, such as excessive gaming and certain sociodemographic factors.

Overall, the present study highlights the complex and multifaceted nature of IGD and social phobia. The findings suggest that sociodemographic factors play an important role in predicting levels of these conditions, and as such, clinicians should take into account the individual’s sociodemographic background when assessing and treating these conditions. Additionally, it is important for clinicians to be aware of the potential negative impact of excessive gaming and work with patients to establish healthy gaming habits.

The findings of this study have important theoretical implications for the field of psychiatry. The diagnostic criteria for Internet Gaming Disorder (IGD) according to the DSM-5 are based on the presence of symptoms such as preoccupation, withdrawal, tolerance, loss of control, giving up other activities, continuation, deception, escape, and negative consequences (21). Our study found that the COVID-19 pandemic has led to an increase in dependency and tolerance towards gaming, which is consistent with the definition of IGD as a progressive loss of control over gaming, tolerance, and withdrawal symptoms. Additionally, our patients reported a cluster of behavioral and cognitive symptoms associated with IGD, including increased social phobia scores, anxiety, nervousness, and loss of self-confidence (22–24).

Although gaming can be a healthy way to relieve stress in the moment, it should not be used as a long-term solution (25, 26). Developing a gaming disorder or other related issues can arise if it becomes a habit rather than a means of relieving pressure (27, 28). Due to the infeasibility of many contingency plans during the COVID-19 pandemic, the maladaptive use of gaming has increased in frequency (15, 29–32). The increased dependency on playing video games during the pandemic observed in our study agrees with reports from other populations (15, 29–32).

We found that increasing playing hours was associated with increasing social phobia scores and aggravating the severity of social phobia (24). Moreover, the onset of playing video games at a younger age is strongly associated with an increased risk of developing social phobia and IGD (33, 34). Our results also revealed that increasing playing time was associated with a higher IGD score, as reported previously (24). However, we observed a worsening in IGD scores in participants who played for a long time due to COVID-19 and in participants with increasing dependency on gaming due to isolation, which is consistent with observations from other studies in different populations (35–38).

This study’s practical implications highlight the need to monitor the increasing prevalence of IGD since the beginning of COVID-19, as it can cause further damage to the psychosocial situation, which is already damaged by the pandemic (1, 5, 13, 30). The results of our study contradict the World Health Organization’s advice to the general population to play video games to reduce COVID-19’s psychosocial effects (10).

### 4.1. Strengths and limitations

There are several strengths and limitations to this study. It is the first study to investigate the impact of the COVID-19 pandemic on gamers in the Arab population, and the sampling method employed ensured that a comparatively representative sample was collected. Additionally, all related factors were assessed using validated questionnaires. However, the limitations of the study include the inherent nature of the data collection tool, which depends on the participant’s ability to recall and complete the self-administered questionnaire. Despite using a validated Arabic version of the questionnaire, there is still a risk of recall bias. Furthermore, the response rate of males who completed the survey was much higher than the response rate of females, indicating that the number of males who are addicted to online gaming may be greater than the number of females. Finally, the study did not examine other variables that may affect IGD and social phobia, such as depression, anxiety, and stress, which may influence the prevalence of IGD (39).

In conclusion, our study highlights the increasing prevalence of IGD during the COVID-19 pandemic in the Arab population and underscores the importance of monitoring its impact on mental health. The findings of this study may have implications for clinicians and policymakers working to mitigate the negative impact of the pandemic on mental health.

### 4.2. Recommendations

The current study highlights the importance of addressing the potential negative impact of COVID-19 on online gaming behavior and the development of IGD among the general population. To mitigate this impact, it is recommended to provide mental health support and counseling to individuals who are at risk of developing IGD due to prolonged isolation and boredom. This can be achieved through various means, such as online counseling, support groups, and educational campaigns to raise awareness about the risks associated with excessive gaming. Moreover, parents and caregivers should monitor and regulate their children’s online gaming activities, especially during prolonged isolation periods, to prevent the development of IGD.

## 5. Conclusion

The present study adds to the growing body of literature on the impact of COVID-19 on mental health and online gaming behavior. Our findings suggest that the COVID-19 pandemic has led to an increase in online gaming activities, and this increase is associated with an increased risk of developing IGD and social phobia symptoms. Therefore, it is crucial to address this issue and provide adequate support to individuals who are at risk of developing IGD. Future studies should investigate the long-term impact of the pandemic on gaming behavior and the effectiveness of different interventions in mitigating the risk of IGD. Overall, this study highlights the importance of recognizing the potential negative impact of COVID-19 on mental health and emphasizes the need for proactive measures to address this issue.

## Data availability statement

The raw data supporting the conclusions of this article will be made available by the authors, without undue reservation.

## Ethics statement

The studies involving human participants were reviewed and approved by Ethics Committee, Faculty of Medicine, Alexandria University. Written informed consent for participation was not required for this study in accordance with the national legislation and the institutional requirements.

## Author contributions

AS and NS contributed equally to the conceptualization, methodology, formal analysis, writing-original draft, review, editing, and data collection. ME, OA, AM, SaS, AR, MM, AZ, and ShS contributed to writing-review and editing. All authors contributed to the article and approved the submitted version.

## Conflict of interest

The authors declare that the research was conducted in the absence of any commercial or financial relationships that could be construed as a potential conflict of interest.

## Publisher’s note

All claims expressed in this article are solely those of the authors and do not necessarily represent those of their affiliated organizations, or those of the publisher, the editors and the reviewers. Any product that may be evaluated in this article, or claim that may be made by its manufacturer, is not guaranteed or endorsed by the publisher.

## Supplementary material

The Supplementary material for this article can be found online at: https://www.frontiersin.org/articles/10.3389/fpsyt.2023.1071764/full#supplementary-material

## Figures and Tables

**Table:** 

COVID-19	Coronavirus disease 2019
SPSS	Statistical Package for the Social Sciences
IGDS-SF9	Nine-item Internet Gaming Disorder Scale-Short Form
SPS	Social Phobia Scale
CDC	Centers for Disease Control and Prevention
US	United States
WHO	World Health Organization
IGD	Internet Gaming Disorder
DSM-5	Diagnostic and Statistical Manual of Mental Disorders, 5th edition
IGA	Internet Gaming Addiction
**Arab nations**	
Egypt	
Syria	
Palestine	
Jordan	
Tunisia	
Iraq	
Morocco	
Algeria	
Sudan	
Libya	
Lebanon	

## Footnotes

^1^ https://www.calculator.net/sample-size-calculator.html
