# Supplementary material for: Gaming behavior disorder and its association with social phobia during COVID-19 pandemic: A cross-sectional study among the young Arabs
Source: Front Psychiatry. 2023 Apr 11;14:1071764. doi: 10.3389/fpsyt.2023.1071764 (PMC10126253; doi:10.3389/fpsyt.2023.1071764)
Supplement: Supplementary file 1 [file Table_1.docx]

**Supplementary file (Survey)**

**Sociodemographic**

First time of gameplay

Before the age of 6 =1

Between 7 and 12 =2

Between 13 and 17 =3

After 18 years old 30 =4

Don’t really remember =5

Daily gameplay hours

Use of substance

Cigarettes

Age

Gender: male= 1 female = 2

**Social phobia scale (SPS)**

| Statement | | Not at all | Slightly | Moderately | Very | Extremely |
| --- | --- | --- | --- | --- | --- | --- |
| 1 | I become anxious if I have to write in front of other people | **0** | **1** | **2** | **3** | **4** |
| 2 | I become self-conscious when using public toilets | **0** | **1** | **2** | **3** | **4** |
| 3 | I can suddenly become aware of my own voice and of others listening to me | **0** | **1** | **2** | **3** | **4** |
| 4 | I get nervous that people are staring at me as I walk down the street | **0** | **1** | **2** | **3** | **4** |
| 5 | I fear I may blush when I am with others | **0** | **1** | **2** | **3** | **4** |
| 6 | I feel self-conscious if I have to enter a room where others are already seated | **0** | **1** | **2** | **3** | **4** |
| 7 | I worry about shaking or trembling when I'm watched by other people | **0** | **1** | **2** | **3** | **4** |
| 8 | I would get tense if I had to sit facing other people on a bus or a train | **0** | **1** | **2** | **3** | **4** |
| 9 | I get panicky that others might see me to be faint, sick or ill | **0** | **1** | **2** | **3** | **4** |
| 10 | I would find it difficult to drink something if in a group of people | **0** | **1** | **2** | **3** | **4** |
| 11 | It would make me feel self-conscious to eat in front of a stranger at a restaurant | **0** | **1** | **2** | **3** | **4** |
| 12 | I am worried people will think my behavior is odd | **0** | **1** | **2** | **3** | **4** |
| 13 | I would get tense if I had to carry a tray across a crowded cafeteria | **0** | **1** | **2** | **3** | **4** |
| 14 | I worry I'll lose control of myself in front of other people | **0** | **1** | **2** | **3** | **4** |
| 15 | I worry I might do something to attract the attention of others | **0** | **1** | **2** | **3** | **4** |
| 16 | When in an elevator I am tense if people look at me | **0** | **1** | **2** | **3** | **4** |
| 17  18  19  20 | I can feel conspicuous standing in a queue | **0** | **1** | **2** | **3** | **4** |
|  | I get tense when I speak in front of other people | **0** | **1** | **2** | **3** | **4** |
|  | I worry my head will shake or nod in front of others | **0** | **1** | **2** | **3** | **4** |
|  | I feel awkward and tense if I know people are watching me | **0** | **1** | **2** | **3** | **4** |

**Nine-item Internet Gaming Disorder Scale-Short Form (IGDS-SF9)**

| Scoring | Never | Rarely | Sometimes | Often | Very Often |
| --- | --- | --- | --- | --- | --- |
| 1. Do you feel preoccupied with your gaming behavior? (Some examples: Do you think about previous gaming activity or anticipate the next gaming session? Do you think gaming has become the dominant activity in your daily life?) | 1 | 2 | 3 | 4 | 5 |
| 2. Do you feel more irritability, anxiety or even sadness when you try to either reduce or stop your gaming activity ? | 1 | 2 | 3 | 4 | 5 |
| 3. Do you feel the need to spend increasing amount of time engaged gaming in order to achieve satisfaction or pleasure? | 1 | 2 | 3 | 4 | 5 |
| 4. Do you systematically fail when trying to control or cease your gaming activity? | 1 | 2 | 3 | 4 | 5 |
| 5. Have you lost interests in previous hobbies and other entertainment activities as a result of your engagement with the game? | 1 | 2 | 3 | 4 | 5 |
| 6. Have you continued your gaming activity despite knowing it was causing problems between you and other people? | 1 | 2 | 3 | 4 | 5 |
| 7. Have you deceived any of your family members, therapists or others because the amount of your gaming activity? | 1 | 2 | 3 | 4 | 5 |
| 8. Do you play in order to temporarily escape or relieve a negative mood (e.g., helplessness, guilt, anxiety)? | 1 | 2 | 3 | 4 | 5 |
| 9. Have you jeopardized or lost an important relationship, job or an educational or career opportunity because of your gaming activity? | 1 | 2 | 3 | 4 | 5 |

**The impact of the epidemic on gaming behavior**

After the outbreak of the COVID-19 pandemic, your behavior at play

Increased greatly =4

Increase slightly =3

It remains as it was before the pandemic =2

Decreased slightly =1

Decreased greatly =0

I think the home isolation period has increased my dependence on electronic games

Totally agree =4

Little agree =3

I do not know =2

I do not agree =1

I don't agree at all =0

I think electronic games are entertainment, relief for the soul during the epidemic

Totally agree =4

Little agree =3

I do not know =2

I do not agree =1

I don't agree at all =0
